# CD1a+ and CD207+ cells are reduced in oral submucous fibrosis and oral squamous cell carcinoma

**DOI:** 10.4317/medoral.23177

**Published:** 2019-12-24

**Authors:** Luan César da Silva, Felipe Paiva Fonseca, Oslei Paes de Almeida, Bruno Augusto Linhares de Almeida Mariz, Márcio Ajudarte Lopes, Raghu Radhakrishnan, Mohit Sharma, Luiz Paulo Kowalski, Pablo Agustin Vargas

**Affiliations:** 1 Department of Oral Diagnosis of Piracicaba Dental School, University of Campinas, Piracicaba, São Paulo, Brazil; 2 Department of Oral Surgery and Pathology, School of Dentistry, University of Minas Gerais, Belo Horizonte, Minas Gerais, Brazil; 3 Department of Oral Pathology and Microbiology, Manipal Academy of Higher Education, Manipal, India; 4 Department of Oral Pathology, Sudha Rustagi College of Dental Sciences and Research, Faridabad, India; 5 Department of Head and Neck Surgery and Otorhinolaryngology, A. C. Camargo Cancer Center, São Paulo, Brazil.

## Abstract

**Background:**

The objective of this study investigated the distribution of immature dendritic cells (DCs), Langerhans cells and plasmacytoid DCs in oral submucous fibrosis (OSMF), OSMF associated with oral squamous cell carcinoma (OSMF-OSCC), oral leukoplakia (OL), and oral squamous cell carcinoma (OSCC).

**Material and Methods:**

Fourteen cases of OSMF, 9 of OSMF-OSCC, 8 of OL¸ 45 of OSCC and 8 of normal epithelium were retrospectively retrieved and their diagnoses confirmed. Immunoreactions against CD1a, CD207 e CD303 were performed and the number of positive cells quantified.

**Results:**

A significant decrease of CD1a+ was found in OSMF (*p*≤0.05), OSMF-OSCC (*p* ≤ 0.01), and OSCC (*p* ≤ 0.001) when compared to normal epithelium. For CD207+ the significance decrease was observed in OSMF-OSCC (*p* ≤ 0.05), and OSCC (*p* ≤ 0.01) when compared with normal epithelium, and in OSMF when compared with OL (*p* ≤ 0.05). There was no significant difference for CD303, but increased in CD303+ was observed in OSCC when compared with normal epithelium.

**Conclusions:**

The decrease in the number of CD1a+ and CD207+ cells may be associate to the development of oral OSCC, and in OPMDs they might be indicators of malignant transformation.

** Key words:**Premalignant lesions, oral submucous fibrosis, oral squamous cell carcinoma, immune response.

## Introduction

Oral squamous cell carcinoma (OSCC) accounts for more than 90% of all oral malignant neoplasms, representing the sixth most common malignancy worldwide (1). In some Asian countries like Sri Lanka, India, Pakistan and Bangladesh, OSCC is even more prevalent (2). This variability in the global incidence of OSCC has been attributed to cultural habits, including the consumption of tobacco, alcohol, and areca nut. In approximately one third of the cases, OSCC may arise from oral potentially malignant disorders (OPMD), such as oral leukoplakia (OL) and oral submucous fibrosis (OSMF).

According to the World Health Organization (WHO), OL is defined as a whitish plaque that cannot be characterized clinically or microscopically as any other entity (3,4). Tobacco smoking has been observed in 70-90% of the patients with OL, (5) and the risk of malignant change varies significantly depending on clinical and pathological features.

**Figure 1:**
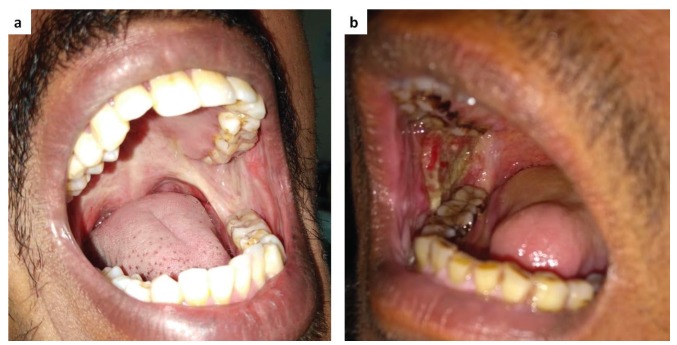
Representative clinical images of patients affected by Oral Submucous Fibrosis (OSMF) and OSMF associated with oral squamous cell carcinoma (OSMF-OSCC). (A) OSMF clinically demonstrating a whiteness and fibrosis of the retromolar area and soft palate. (B) OSMF-OSCC with extensive ulcerative areas.

OSMF represents a public health problem, mainly in India. Previous studies have associated OSMF with use of areca nut, which is potentially carcinogenic; however, the biological mechanisms involved are not well established (6,7). The most common malignant neoplasm in South and Southeast Asia is OSMF associated with OSCC (OSMF-OSCC) (8,9). OSMF is a fertile soil for malignancy and various grades of OSCC do arise in background of OSMF (Fig. 1). Moreover, malignancy occurs at an accelerated pace in OSMF due to convergence of several pathways and mechanisms (10). Additionally, arecoline a component of arecanut has been shown to induce genomic instability by producing aberrances of mitotic spindle assembly and spindle assembly checkpoints (11). It seems that the OSCC arising from OSMF and that arising from OL carry widely varying prognostic implications, and there is an imperative need to study the same.

The immune system has an important role in regulating OPMD and frankly invasive lesions. Dendritic cells (DCs) are antigen-presenting cells responsible for starting the immune response mediated by B and T lymphocytes (12). An adequate immune response protects the mucosa from malignant transformation (13). The distribution of DCs has been studied in several lesions for their ability to recognize precursor malignant cells and to destroy them. We have previously demonstrated a reduction of DC in lip SCC and in actinic cheilitis if compared to normal lip mucosa (14), as well as in OSCC if compared to normal oral mucosa (13), however, difference in the distribution of DC between OSCC and OSMF-OSCC is unknown. Therefore, in the current study we attempted to determine the distribution of immature DCs, Langerhans cells and plasmacytoid DCs (pDCs) in OSMF, OSMF-OSCC, OL, and OSCC.

## Material and Methods

The study was approved by the ethical committee of the *Pi*racicaba Dental School (protocol: 69395817.8.0000.5418). This study includes cases of OSMF (n=14) and OSMF-OSCC (n=9) retrieved from the files of the Department of Oral Pathology and Microbiology of the Manipal Academy of Higher Education (Manipal, Udupi, Karnataka, India) and another 8 and 45 cases of OL (all the cases presenting epithelial dysplasia) and OSCC from the files of two institutions: *Pi*racicaba Dental School, University of Campinas, Brazil; and AC Camargo Cancer Center, São Paulo, Brazil. Clinical information such as gender, age and risk factors was collected from patients’ records. Eight cases morphologically normal epithelial tissue adjacent to mucoceles were used as control.

Two independent oral pathologists reviewed the original 5µm histological sections stained with hematoxylin and eosin (H&E) of all cases and confirmed the diagnoses. Immunohistochemistry was carried out on 3µm sections of the original formalin-fixed, paraffin-embedded tissues. Slides were dewaxed with xylene and rehydrated in descending ethanol solutions (absolute, 90%, 80% and 70%). Antigen retrieval was performed using Tris/EDTA (pH: 8.0) and the endogenous peroxidase activity was blocked using 10% hydrogen peroxide. Slides were washed in PBS buffer (pH 7.4) and incubated at room temperature for 120 min with the antibodies: CD207 (Clone EPR15863; Abcam, USA) CD303 (Clone 124B3.13, 1:50; Dendritics, USA) and CD1a (Clone 010, RTU; DAKO Co., USA).

All slides were subsequently exposed to an avidin–biotin complex containing appropriate secondary antibody (Vectastain Elite ABC kit; Vector Laboratories, Burlingame, CA, USA). The diaminobenzidine tetrahydrochloride (Sigma, St. Louis, MO, USA) was used for the immunochemical reactions according to the manufacturer’s instructions; the samples were counterstained with Carazzi haematoxylin.

Dendritic cells were manually quantified by one previously trained observer. Ten sequential fields randomly were selected and photographed at 200x magnification for positive cell counts. The quantification was performed based on the methodology described by Pellicioli *et al* 2017. DCs were quantified in the epithelial tissue in the OSMF and OL groups, and in the epithelial infiltrative component and connective tissue from OSMF-OSCC and OSCC groups.

The software GraphPad Prism (version 5.0, San Diego, California, USA) was used for the statistical analysis. Data were submitted to analysis of variance (ANOVA) and Tukey tests at a significance level of *p* < 0.05.

## Results

Clinical data are shown in Table 1. Males predominated in all lesions. Mean age for the OSCC group (58.5 years) was higher than in the OSMF-OSCC group (36.5 years). Use of isolated tobacco was reported in cases of OL and OSCC groups.

Table 2 shows the distribution of DCs for all groups. We demonstrate a significance reduction for CD1a+ and CD207+. DCs were identified as ramified cells in normal/neoplastic epithelium and connective tissues highlighted by the specific antibodies staining the cell membrane.

**Figure 2:**
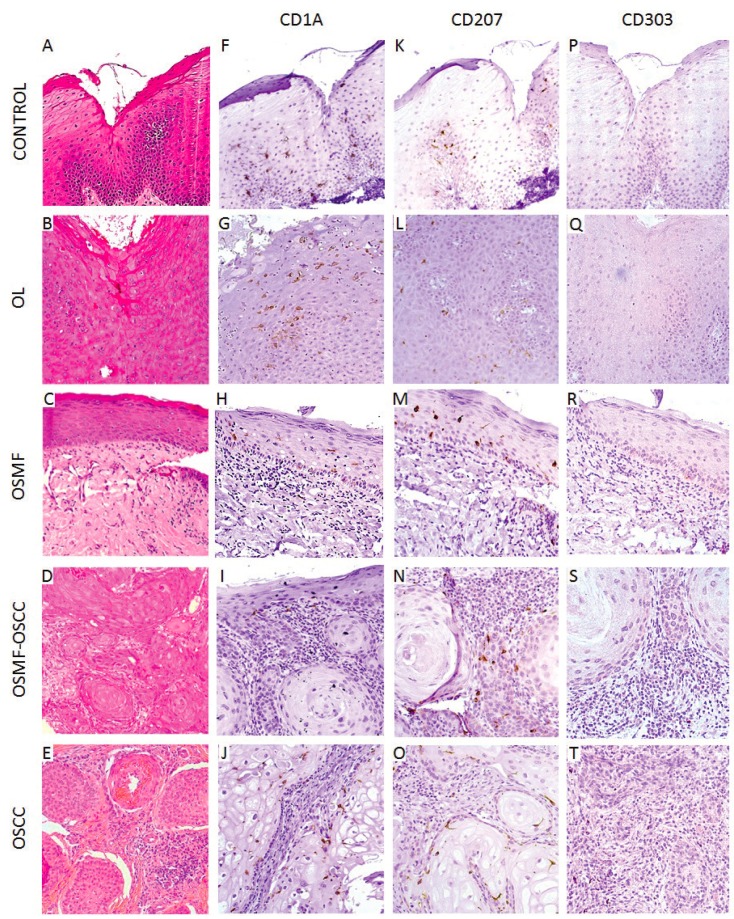
Histopathological features and immunohistochemical detection of immature dendritic cells (DCs) and Langerhans cells (LCs) of control group (mucocele), oral leukoplakia (OL), oral submucous fibrosis (OSMF), OSMF associated with oral squamous cell carcinoma (OSMF-OSCC) and oral squamous cell carcinoma (OSCC). (A) Histologically normal oral epithelium without dysplastic alterations (hematoxylin and eosin – H&E; 200x). (B) Microscopic features of OL (H&E; 200x). (C) Microscopic features of OSMF demonstrating submucosal and justaepithelial deposition of collagenated connective tissue (H&E; 200x). (D) OSFM-OSCC with infiltrative features and cellular atipia (H&E; 200x). (E) OSCC demonstrating infiltrative tumor nests (H&E; 200x). (F) Immunohistochemical expression of CD1a+ cells in the epithelium tissue of the control group (mucocele). (G) OL group. (H) OSMF. (I) OSMF-OSCC. (J) OSCC. (K) CD207+ cells in the control group (mucocele). (L) OL group. (M) OSMF. (N)OSMF-OSCC. (O) OSCC. There was a remarkable decrease of CD1a+ and CD207+ cells in the OSCC group.

**Figure 3:**
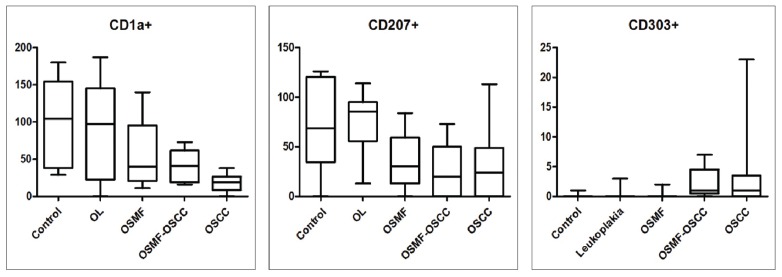
Distribution of immature dendritic cells (DCs) (CD1a+), Langerhans cells (CD207+) and plasmacytoid DCs (CD303+) in the epithelial compartment of the control group, oral leukoplakia (OL), and oral submucous fibrosis (OSMF), and in the tumor nests of the OSMF associated with oral squamous cell carcinoma (OSMF-OSCC) and oral squamous cell carcinoma (OSCC) group. Immature DCs were significantly decreased in OSMF, OSMF-OSCC e OSCC when compared to the control group, and LC also were significantly decreased in OSMF-OSCC e OSCC when compared to the control group.

Fig. 2 and Fig. 3 shows a decrease in the distribution of CD1a+ and CD207+ cells in the OSCC group when compared with the normal epithelium used as control. CD1a+ and CD207+ cells were observed throughout the epithelium, while, in the connective tissue, they were more prevalent in the tumor nests of the OSMF-OSCC and OSCC lesions. seen in OSMF when compared with OL (*p* ≤ 0.05).

**Table 1:**
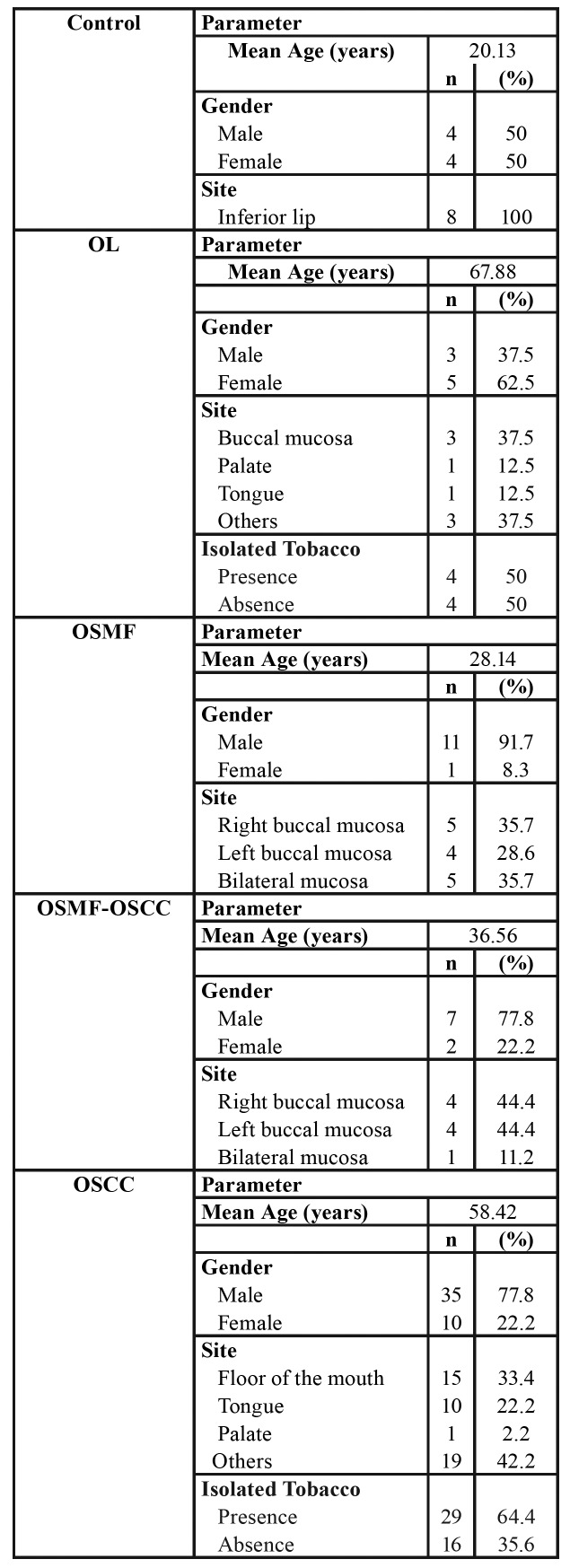
Clinical features of control, OL, OSMF, OSFM-OSCC, and OSCC groups.

A slight increase in the amount of DCs was observed in CD303. The concentration of CD303+ cells was increased in all groups when compared to the control group (normal epithelium). The mean of CD303+ (Table 2) was 0.13 in the control group, while in the other groups were found values such as 0.38 (OL), 0.21 (OSMF), 2.22 (OSMF-OSCC) and 2.73 (OSCC). However, no statistical difference was found for CD303 for all groups studied.

Table 3 shows the significant decrease in CD1a+ and CD207+. We observed which in CD1a was differences in OSMF (p≤0.05), OSMF- OSCC (*p* ≤ 0.01), and OSCC (*p* ≤ 0.001) when compared to normal epithelium. For CD207 in OSMF-OSCC (*p* ≤ 0.05), and OSCC (*p* ≤0.01) when compared to normal epithelium, and this reduction was also seen in OSMF when compared with OL (*p* ≤ 0.05).

**Table 2:**
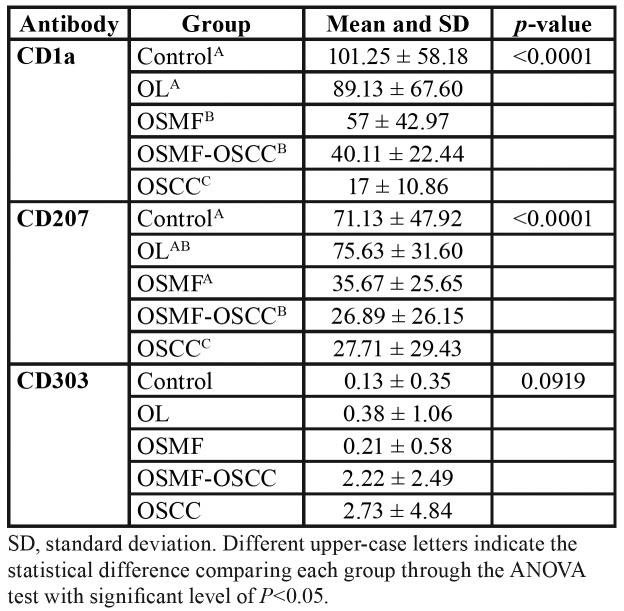
Quantification of positive cells for all the antibodies in each group.

**Table 3:**
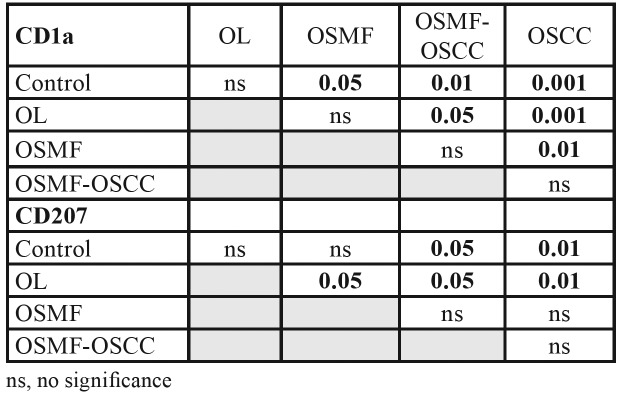
Comparison between all the groups for CD1a and CD207 antibodies by Tukey analysis.

## Discussion

The role of the immune system cells in oral diseases have been widely investigated (13-15). Such cells, especially the DCs, are responsible for recognizing the antigen, processing it, and presenting it to T cells, essential steps to regulate the innate and adaptive immunity systems (16). Our study investigated the distribution profile of the DCs in OSMF, OSMF-OSCC, OL, and OSCC and revealed a significant decrease of CD1a+ and CD207+ cells in oral cancer and OSMF, but not for CD303 positive cells.

Several DC subtypes are present in the oral mucosa and the concentration of these cells may vary according to the anatomical topography (17). Our findings show a decrease in CD1a+ and CD207+ cells in all the groups studied when compared to the normal epithelium, except in OL for CD1a+. Supporting this finding, some studies have shown which an imbalance in DCs in OPMD and frankly invasive lesions can result in less activity of the immune response.

Among the markers available for detecting DCs are S100, CD1a, CD83, CD207, CD208, CD80, CD11c, CD86, CD303 and HLA-DR (16,18). In our study, CD1a, CD207, and CD303 were selected based on previous studies investigating different malignant neoplasms and the biological behavior of such markers (13,14). CD1a and CD207 are effective in identifying immature DCs and Langerhans cells. CD303 is used to identify pDCs, which are resident in lymphoid or non-lymphoid organs and are responsible for the production of type I interferon (19,20).

A decrease in the number of DCs in OL has been associated with a higher risk of malignant transformation (13,16). Additionally, in OL cases DCs decreases occur in the presence of smoking habit and increases occur in larger lesions and in the presence of dysplasia (21). Certainly, our results show higher CD207+ expression in OL when compared to controls indicating intact DCs function in this stage (Table 2, Fig. 2).

A reduction in the distribution of DCs was observed in the OSCC when compared with the OL. Such reduction resulted in a decrease in the presentation of antigens, a condition that may favor the development and the progression of OSCC (16). This analysis might allows better understand the role of malignant transformation.

CD303+ cells have been associated with organism defense (13). In our study, a gradual increase in CD303+ cells was observed for OSMF, OL, OSMF-OSCC, and OSCC, respectively, when compared to the control. This increase in the CD303+ cells might be related to the immune defense mechanism.

The antitumor immune responses may vary according to the individuals’ health status (22). It is well established that OSMF is associated with the chewing of areca nuts; whereas OL is strongly associated with the use of isolated tobacco (5,7,23). When compared to non-smokers, smokers revealed a smaller number of DCs in their oral mucosa, a condition that can reduce the individual’s immune response (15).

CD207 immunoreactivity was lower in OSMF-OSCC than in conventional OSCC, suggesting that arecanut might induce a decrease in DCs to a greater extent than that induced by isolated tobacco. The expression of the CD207 receptor in DCs evidences the antigen is effectively coupled with MHC-I and MHC-II, a condition that activates the CD8+ and CD4+ T cells (24); therefore, the down-regulation of this receptor in DCs might indicate a suppression of the T-cell response. Further studies with a greater number of samples and different molecular tests are needed to investigate this hypothesis and verify our findings.

In conclusion, the decrease in the number of CD1a+ and CD207+ cells may be associate to the development of OSCC, and in OPMDs they might be indicators of malignant transformation. Our preliminary results indicate that arecanut chewing may produce a greater suppression of immune cell function when compared to cigarette smoking alone. Further studies on these cellular alterations are needed for a better understanding of the differential malignant transformation of OPMDs like OL and OSMF, and their impact on the disease pathogenesis and prognosis.
